# Improvement of insulin signalling rescues inflammatory cardiac dysfunction

**DOI:** 10.1038/s41598-019-51304-8

**Published:** 2019-10-15

**Authors:** Isehaq Al-Huseini, Masayuki Harada, Kiyoto Nishi, Dat Nguyen-Tien, Takeshi Kimura, Noboru Ashida

**Affiliations:** 1 Department of Cardiovascular Medicine, Graduate School of Medicine, Kyoto University, Kyoto, Japan; 2 Present Address: Department of Physiology, College of Medicine and Health Sciences, Sultan Qaboos University, Muscat, Oman; 3 Present Address: Mitochondria and Metabolism Center, Department of Anaesthesiology and Pain Medicine, University of Washington, Seattle, WA98109 USA; 4 Present Address: Department of Molecular Immunology and Inflammation, Research Institute, National Center for Global Health and Medicine, 1-21-1 Toyama, Shinjuku-ku, Tokyo 162-8655 Japan

**Keywords:** Cell signalling, Cell signalling, Cardiovascular diseases, Cardiovascular diseases

## Abstract

Inflammation resulting from virus infection is the cause of myocarditis; however, the precise mechanism by which inflammation induces cardiac dysfunction is still unclear. In this study, we investigated the contribution of insulin signalling to inflammatory cardiac dysfunction induced by the activation of signalling by NF-κB, a major transcriptional factor regulating inflammation. We generated mice constitutively overexpressing kinase-active IKK-β, an essential kinase for NF-κB activation, in cardiomyocytes (KA mice). KA mice demonstrated poor survival and significant cardiac dysfunction with remarkable dilation. Histologically, KA hearts revealed increased cardiac apoptosis and fibrosis and the enhanced recruitment of immune cells. By molecular analysis, we observed the increased phosphorylation of IRS-1, indicating the suppression of insulin signalling in KA hearts. To evaluate the contribution of insulin signalling to cardiac dysfunction in KA hearts, we generated mice with cardiac-specific suppression of phosphatase and tensin homologue 10 (PTEN), a negative regulator of insulin signalling, in the KA mouse background (KA-PTEN). The suppression of PTEN successfully improved insulin signalling in KA-PTEN hearts, and interestingly, KA-PTEN mice showed significantly improved cardiac function and survival. These results indicated that impaired insulin signalling underlies the mechanism involved in inflammation-induced cardiac dysfunction, which suggests that it may be a target for the treatment of myocarditis.

## Introduction

Myocarditis is a critical disease characterized by significant cardiac dysfunction and can eventually lead to inflammatory dilated cardiomyopathy (DCMi)^1–3^. The pathological cause is the inflammation of cardiac muscle, which is induced mainly by viral infection^4,5^; however, the mechanism by which inflammation induces cardiac dysfunction is still unclear. For example, the involvement of nuclear factor-κB (NF-κB), which is activated in viral myocarditis^6^, in the progression of myocarditis has not been adequately assessed.

NF-κB is part of a family of transcription factors that plays a critical role in inflammation. NF-κB generally exists as a homo- or heterodimer in the cytosol that is bound to the inhibitor of κB (IκB)^7^. In response to a wide variety of stimuli, including inflammatory cytokines, IκB is phosphorylated and degraded via the ubiquitin pathway, which is followed by the translocation of NF-κB to the nucleus and the activation of the transcription of inflammatory cytokines. The serine phosphorylation of IκB is mediated by a large multi-unit complex containing two catalytic subunits, IKKα and IKKβ, and the regulatory subunit IKKγ, also known as NEMO^8^. Of these subunits, IKKβ is the essential kinase that mediates IκB phosphorylation^8^.

Coxsakievirus and lipopolysaccharide have been widely used to induce myocarditis in mice model through the activation of inflammatory signalling^9,10^. Moreover, Maier *et al*. reported that activation of IKKβ/ NF-κB in the cardiomyocytes induced the phenotype of DCMi in mice^6,11^. As indicated in those reports, there is consensus on the involvement of the inflammation in the progression of DCMi; however, targeting NF-κB signalling with immune suppressive drugs is not always effective for treatment of DCMi^12,13^. It might be because suppression of NF-κB signalling can be detrimental for the heart, since NF-κB has protective functions like ati-apoptotosis and antioxidant^14,15^. Therefore, exploring for possible involvement of other signallings in the progression of DCMi will help to understand the underlying mechanism and find effective treatment for this disease.

Pro-inflammatory cytokines regulated by NF-κB have been reported to induce cardiac insulin resistance characterized by an impaired insulin effect in glucose transport in cardiac muscle^16^. In fact, cardiac insulin resistance is known to induce DCM independently from inflammation^17–19^, however its contributions during the progression of DCMi is still not fully understood. Recently it has been reported that metformin, a widely-used anti-diabetic drug, attenuated myocarditis induced by endotoxin in mice^20^ and associated with lower mortality in ambulatory patients with diabetes and heart failure^21^. Such interaction between insulin and inflammatory signalling and their implications in progression of DCM encouraged us to investigate the contribution of insulin signalling in DCMi.

To explore the underlying mechanism of DCMi, we generated mice overexpressing kinase-active IKKβ in cardiomyocytes (KA mice). The mice suffered from severe cardiac dysfunction and died at an early age. Based on previous reports showing that insulin signalling contributes to the mechanism underlying DCM^22–24^ and is impaired by the activation of IKKβ^25–28^, we analysed insulin signalling and found that it was impaired in the hearts of KA mice. Furthermore, the rescue of insulin signalling by cardiac-specific suppression of PTEN (phosphatase and tensin homologue 10), a negative regulator of insulin signalling^29,30^, in KA mice improved cardiac function and survival. These results indicate that impaired insulin signalling is involved in the mechanism underlying inflammation-induced cardiac dysfunction, at least in part, and implicates a possible application of diabetes medicine for the improvement of insulin resistance in the treatment of myocarditis or DCMi.

## Results

### Generation of mice with cardiomyocyte-specific IKKβ activation

**Figure 1:**
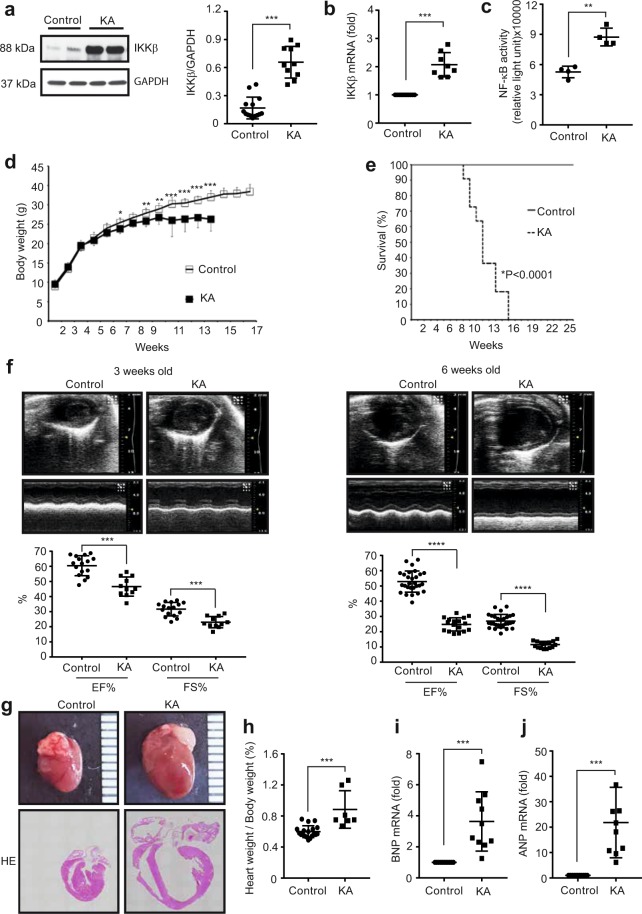
Constitutive IKKβ activation in cardiomyocytes induces cardiac dysfunction. (**a**) Representative Western blots and densitometric analysis of IKKβ/GAPDH in heart lysates. Scatter dot plots represent the mean ± SD, ***P < 0.001 ((*t*-test, n = 7 control, n = 8 KA). (**b**) Results of qRT-PCR of the expression of IKKβ mRNA isolated from heart tissue and normalized to the Rn18s mRNA level. Scatter dot plots represent the mean ± SD, ***P < 0.001 (*t*-test, n = 11 control, n = 8 KA). (**c**) NF-κB activity in heart tissue. Scatter dot plots represent the mean ± SD, **P < 0.01 (*t*-test, n = 4). (**d**) Body weight ± SD of control and KA littermate mice at the indicated ages, *P < 0.05, **P < 0.01, ***P < 0.001 (two-way repeated measure ANOVA, n = 13 control, n = 11 KA). (**e**) The Kaplan-Meier survival curve of control and KA mice (n = 13 control, n = 11 KA) ****P < 0.0001. (**f**) The cardiac function of control and KA mice at three and six weeks of age as determined by echocardiography. Scatter dot plots represent the mean ± SD, ***P < 0.001, ****P < 0.0001 (*t*-test, n = 16 control, n = 11 KA at the age of three weeks; n = 28 control, n = 17 KA at the age of six weeks). (**g**) Representative images of hearts (upper images) and heart cross-sections stained with haematoxylin and eosin (lower images) from control and KA mice at the age of six weeks. (**h**) Heart weight/body weight ratio of control and KA mice at the age of six weeks. Scatter dot plots represent the mean ± SD, ***P < 0.001 (*t*-test, n = 19 control, n = 7 KA). (**i,j**) Results of qRT-PCR for the expression of BNP and ANP mRNA isolated from heart tissue from control and KA mice at the age of six weeks and normalized to the Rn18s mRNA level. Scatter dot plots represent the mean ± SD, ***P < 0.001 (*t*-test, n = 11 control, n = 10 KA). qRT-PCR: quantitative real time-polymerase chain reaction; NF-κB: nuclear factor-κB; KA: kinase-active IKKβ; SD: standard deviation; GAPDH: glyceraldehyde 3-phosphate dehydrogenase; BNP: brain natriuretic peptide; ANP: atrial natriuretic peptide.

IKKβ is an essential kinase in the canonical pathway of NF-κB, and it activates NF-κB through the phosphorylation and degradation of IκB^31^. To assess the role of NF-κB signalling in inflammation-induced cardiac dysfunction, we generated mice with constitutively overexpressed IKKβ (KA) with selective activity in cardiomyocytes and compared them with the littermate control mice. The results were similar to those observed in a previously reported myocarditis mouse model^11^, but we chose not to use a drug-inducible system to avoid the possible effect of doxycycline on the heart. Mice were born according to Mendelian frequencies, and Western blot and RT-PCR analysis indicated that IKKβ was successfully overexpressed in the heart (Fig. 1a,b). Next, we examined the activity of NF-κB by measuring the active form of NF-κB (p65), which binds to a specific DNA sequence, in control and KA heart tissues. The assay results showed a significant increase in NF-κB activity in KA hearts compared to that in control hearts (Fig. 1c). These results indicate that IKKβ-NF-κB signalling was successfully activated in KA hearts.

### Cardiomyocyte-specific IKKβ activation causes cardiac dysfunction

Compared with the littermate control mice, KA mice showed a significant decrease in body growth starting at the age of six weeks and demonstrated progressive mortality between eight and fourteen weeks (Fig. 1d,e). We performed echocardiography to assess cardiac function at the ages of three weeks and six weeks and found that the ejection fraction (EF%) and fractional shortening (FS%) were significantly decreased in three-week-old KA mice and worsened at the age of six weeks (Fig. 1f, Supplemental video 1). Moreover, the KA heart showed remarkable dilation, with thinning in both the ventricle and atrial walls (Fig. 1g). At the age of six weeks, KA mice showed a significant increase in the heart weight/body weight ratio (Fig. 1h). Furthermore, mRNA expression of atrial natriuretic peptide (ANP) and brain natriuretic peptide (BNP), which are diagnostic and predictive markers for heart failure, were significantly increased in the hearts of KA mice when compared with that in the hearts of littermate control mice (Fig. 1i,j). These results indicate that the overexpression of constitutively active IKKβ in cardiomyocytes induced cardiac dysfunction, which led to high mortality.

### Cardiomyocyte-specific IKKβ activation induces insulin resistance through the phosphorylation of IRS-1

**Figure 2:**
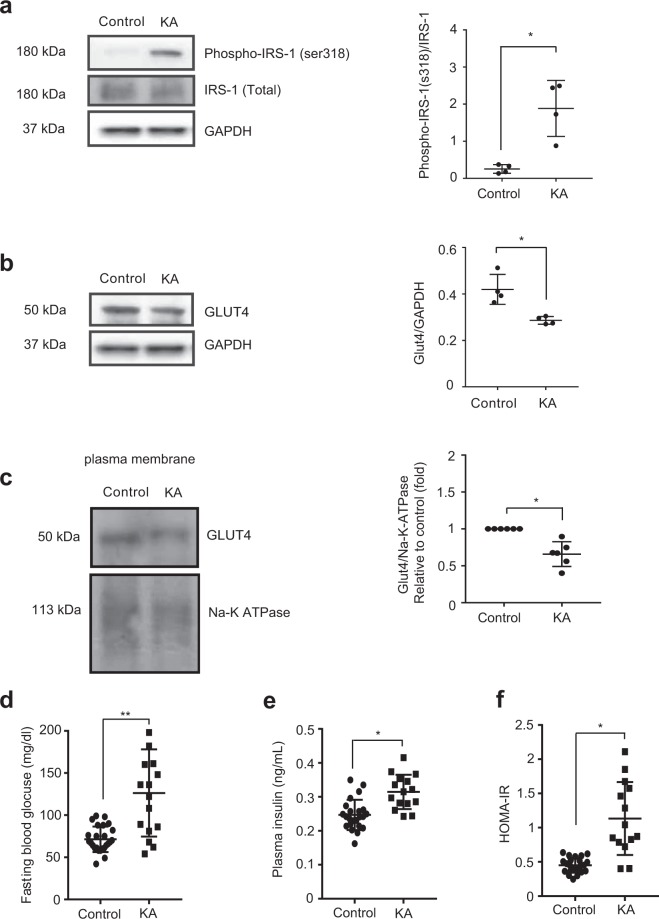
Activation of IKKβ in cardiomyocytes induces IR. (**a**) Representative Western blots and densitometric analyses of phospho-IRS-1^ser318^/total IRS-1 in heart lysates. Scatter dot plots represent the mean ± SD, *P < 0.05 (*t*-test, n = 4 control, n = 4 KA). (**b**) Representative Western blots and densitometric analyses of total Glut4/GAPDH in whole heart lysate. Scatter dot plots represent the mean ± SD, *P < 0.05 (*t*-test, n = 4 control, n = 4 KA). (**c**) Representative Western blots and densitometric analyses of plasma membrane fraction of Glut4/Na-K-ATPase (relative to control) in heart tissue from control and KA mice at the age of six weeks. Scatter dot plots represent the mean ± SD, *P < 0.05 (*t*-test, n = 6 control, n = 6 KA). (**d**) Results of fasting blood glucose (FBG) measurements after 16 hours of fasting at the age of six weeks. Scatter dot plots represent the mean ± SD, *P < 0.05, **P < 0.01 (*t*-test, n = 16 control, n = 17 KA). (**e**) Results of plasma insulin measurements after 16 hours of fasting at the age of six weeks. Scatter dot plots represent the mean ± SD, *P < 0.05 (*t*-test, n = 16 control, n = 16 KA). (**f**) HOMA-IR results (FBG × plasma insulin/40). Scatter dot plots represent the mean ± SD, *P < 0.05 (*t*-test, n = 16 control, n = 16 KA). Scatter dot plots represent the mean ± SD, *P < 0.01 (*t*-test, n = 6 control, n = 6 KA). KA: kinase-active IKKβ; SD: standard deviation; GAPDH: glyceraldehyde 3-phosphate dehydrogenase; IRS-1: insulin receptor substrate-1.

Several reports indicated that the inhibition of insulin signalling induces a deleterious effect on heart function^32–34^. For example, Yujuan Qi *et al*. reported that the double knockout of insulin receptor substrate-1 (IRS-1) and IRS-2 is sufficient to induce dilated cardiomyopathy in mice^35^. Interestingly, IKKβ has been reported to induce insulin resistance (IR) and the diminished insulin-dependent stimulation of glucose uptake^25–28,36^, which is known to induce cardiac dysfunction by itself^22–24,37^. Therefore, we hypothesized that impaired insulin signalling in the KA heart might contribute to the mechanism underlying cardiac dysfunction. To investigate the underlying mechanism, we examined the phosphorylated form of insulin receptor substrate-1 (IRS-1) and Glut4 trafficking. IKKβ has been reported to induce insulin resistance through IRS-1 phosphorylation and inactivation, and this leads to the downregulation of Glut4, a regulator of glucose transport into the cell^38–44^. Phosphorylation of IRS-1 not always induce insulin resistance, depending on Ser or Tyr, and phosphorylation site^43^. Therefore we examined the phosphorylated forms of IRS-1 at ser^318^, which is reported to inhibit IRS-1 activation and subsequently suppress insulin sensitivity^45^, and Glut4 trafficking. We found a significant increase in the level of IRS-1 phosphorylated at Ser^318^ and a decrease of Glut4 in plasma membrane fraction from KA hearts (Fig. 2a–c). It is noteworthy that total expression of Glut4 in whole cell lysate is also decreased in KA heart, which would be due to the suppressing effect of NF-κB on Slc2a4 gene, which encodes Glut4^46^.

To characterize the metabolic phenotype, we checked the fasting blood glucose (FBG) at the age of six weeks after 16 hours of fasting. Interestingly, FBG was significantly elevated in KA mice compared with that in control mice (Fig. 2d). Furthermore, plasma insulin was significantly increased in KA mice compared with that in control mice (Fig. 2e). Consistent with these results, the results of the homeostatic model assessment of insulin resistance (HOMA-IR) were significantly increased in KA mice (Fig. 2f).

These results indicate that the constitutive activation of IKKβ in cardiomyocytes impairs insulin signalling through phosphorylation and inhibition of IRS-1.

### Recovery of impaired insulin signalling in KA hearts by the suppression of PTEN

There are several ways to improve insulin signalling by genetic modification, including the overexpression of PI3K or Akt, but the knock-out of a gene is better than its overexpression because it allows for the evaluation of its effect to be more precise. Therefore, to investigate the contribution of insulin signalling to cardiac dysfunction observed in KA hearts, we generated mice with the cardiac-specific heterozygous knock-out of phosphatase and tensin homologue 10 (PTEN) in the KA mouse background (KA-PTEN). PTEN functions as a negative regulator of insulin signalling^47,48^, and its suppression activates insulin signalling at the post-receptor level, including at the level of IRS-1^30,48–51^. Therefore, the impairment of insulin signalling in KA hearts was expected to be reduced in KA-PTEN hearts.

**Figure 3:**
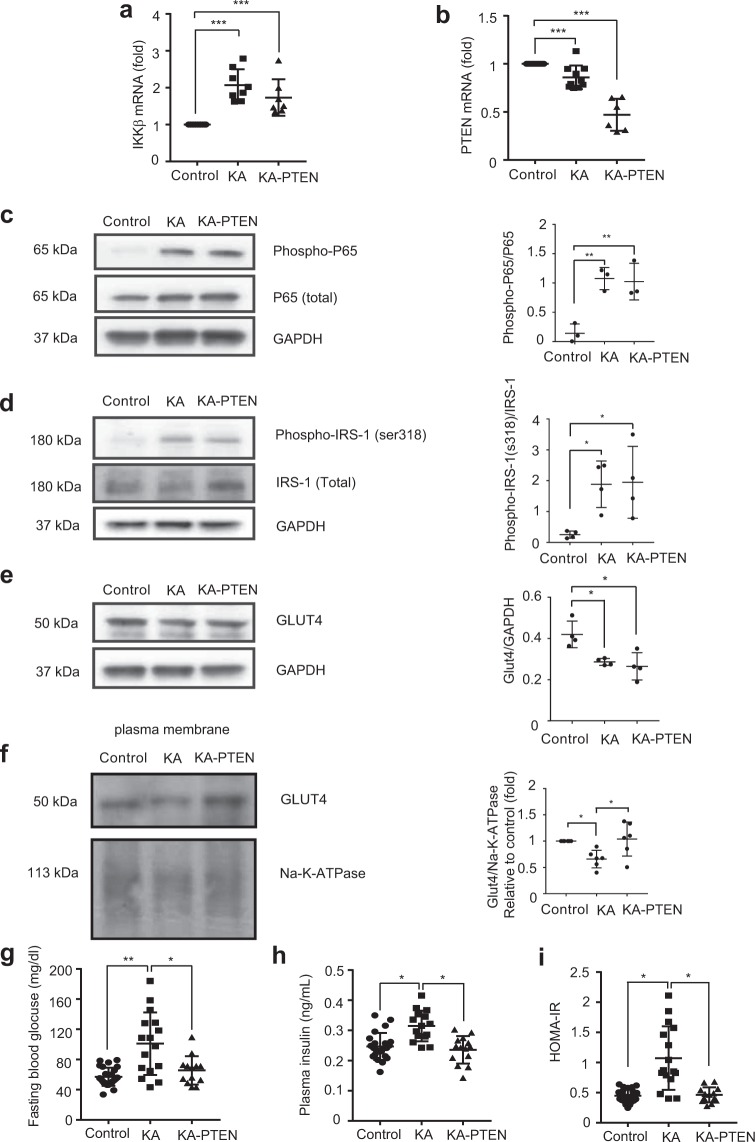
Recovery of impaired insulin signalling in KA hearts by PTEN suppression. (**a,b**) Results of qRT-PCR for the expression of IKKβ and PTEN mRNA isolated from the hearts of control, KA, and KA-PTEN mice at the age of six weeks old and normalized to the Rn18s mRNA level. Scatter dot plots represent the mean ± SD, ***P < 0.001 (One-way ANOVA, n = 11 control, n = 8 KA, n = 7 KA-PTEN). (**c**) Representative Western blots and densitometric analyses of phospho-p65/p65 in heart lysates from control, KA and KA-PTEN mice at the age of six weeks. Scatter dot plots represent the mean ± SD, **P < 0.01 (one-way ANOVA, n = 3 control, n = 3 KA, n = 3 KA-PTEN). Full-length blots are presented in Supplementary Fig. 1. (**d**) Representative Western blots and densitometric analyses of phospho-IRS-1^ser318^/total IRS-1 in heart lysates from control, KA and KA-PTEN mice at the age of six weeks. Scatter dot plots represent the mean ± SD, *P < 0.05 (one-way ANOVA, n = 4 control, n = 4 KA, n = 4 KA-PTEN). Full-length blots are presented in Supplementary Fig. 2. (**e**) Representative Western blots and densitometric analyses of total Glut4/GAPDH in whole heart lysate. Scatter dot plots represent the mean ± SD, *P < 0.05 (one-way ANOVA, n = 4 control, n = 4 KA). Full-length blots are presented in Supplementary Fig. 2. (**f**) Representative Western blots and densitometric analyses of Glut4/Na-K-ATPase levels in heart lysates from control, KA and KA-PTEN mice at the age of six weeks. The band intensity of each protein is normalized with that of Na + K + ATPase, and the values are expressed as fold increase relative to control. Scatter dot plots represent the mean ± SD, *P < 0.05 (one-way ANOVA, n = 6 control, n = 6 KA, n = 6 KA-PTEN). Full-length blots are presented in Supplementary Fig. 3. (**g**) FBG results determined after 16 hours of fasting at the age of six weeks. Scatter dot plots represent the mean ± SD, *P < 0.05, **P < 0.01 (one-way ANOVA, n = 25 control, n = 16 KA, n = 17 KA-PTEN). (**h**) Results of plasma insulin determined after 16 hours of fasting at the age of six weeks. Scatter dot plots represent the mean ± SD, *P < 0.05 (one-way ANOVA, n = 26 control, n = 16 KA, n = 13 KA-PTEN). (**i**) HOMA-IR results (FBG x plasma insulin/40). Scatter dot plots represent the mean ± SD, *P < 0.05 (one-way ANOVA, n = 16 control, n = 16 KA, n = 13 KA-PTEN). qRT-PCR: quantitative real time-polymerase chain reaction; KA: kinase-active IKKβ; KA-PTEN: kinase-active IKKβ with suppressed PTEN; SD: standard deviation; GAPDH: glyceraldehyde 3-phosphate dehydrogenase; IRS-1: insulin receptor substrate-1; HOMA-IR: Homeostatic Model Assessment of Insulin Resistance; FBG: fasting blood glucose. Note: the results for the control and KA groups presented in Figs 2 and 3 are the same.

KA-PTEN mice were born according to Mendelian frequencies and showed normal growth with an absence of premature mortality. PTEN was successfully suppressed in the hearts of KA-PTEN mice, while IKKβ activation was retained (Fig. 3a,b). Moreover, PTEN mRNA expression showed a modest but significant decrease in the hearts of KA mice compared with that in hearts of the littermate control mice. Previously, Vasudevan *et al*. have reported about the suppression of PTEN expression by NF-κB^52^. The change in our study is only modest compared to the report possibly due to the difference in cell types, however this report would explain it. Western blot analysis of heart tissue from KA-PTEN mice confirmed the upregulation of NF-κB activity as indicated by the significant increase of phosphorylated p65, a major subunit of NF-κB and an indicator of its activity (Fig. 3c). Phosphorylated IRS-1at ser318 was retained in both KA and KA-PTEN hearts due to constitutive activation of IKKβ in both types of mice (Fig. 3d). Meanwhile, Glut4 in the plasma membrane was remarkably upregulated in KA-PTEN hearts compared to that in KA hearts (Fig. 3e,f), despite of similarly suppressed total Glut4 expression due to the activated NF-κB^46^ in KA and KA-PTEN.

Of note, Akt signalling was examined by Western blot in heart tissue, however the expression of both phosphorylated forms and total protein of Akt and its downstream target AS160 were not consistent (Supplemental Fig. 4). Considering of the clear results in phosphorylation of IRS-1, we suppose it is because Akt is regulated by many signalling including integrin or cytokines other than insulin, which is different situation from IRS-1. It possibly masked the clear difference in phosphorylation of Akt or AS160 induced by insulin signalling, especially in the *in vivo* samples from whole heart.

Furthermore, we investigated whether the activation of insulin signalling by the suppression of PTEN in the heart had an impact on the levels of blood glucose and insulin. Consistent with the result for Glut4, KA-PTEN mice showed significant decreases in FBG and plasma insulin levels when compared with KA mice (Fig. 3g,h). Moreover, HOMA-IR was reduced significantly in KA-PTEN mice compared with KA mice (Fig. 3i). These results indicate that impaired insulin signalling was successfully recovered by PTEN suppression.

### Suppression of PTEN improves cardiac function in KA hearts

**Figure 4:**
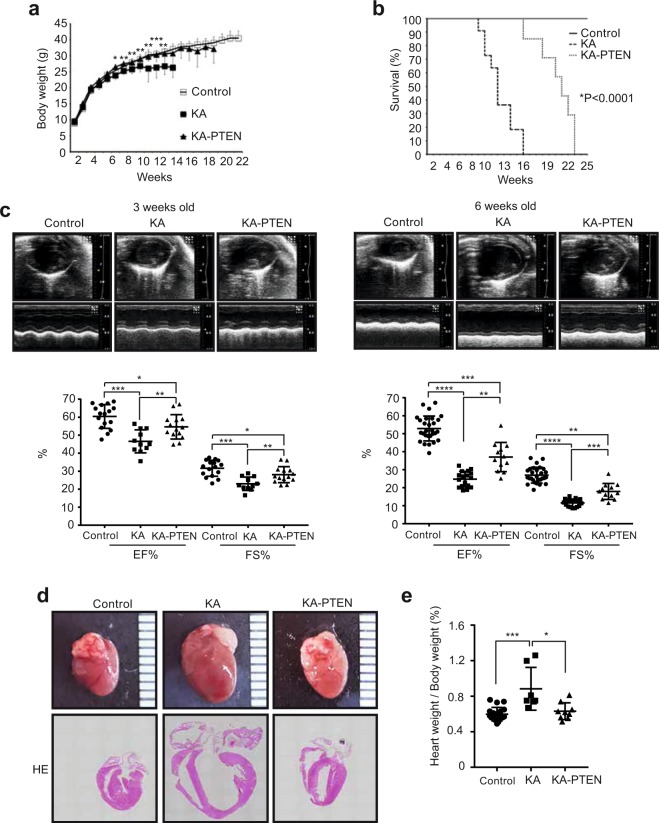
PTEN suppression improved cardiac function in KA mice. (**a**) Body weight ± the SD in control, KA, and KA-PTEN mice at the indicated ages, *P < 0.05, **P < 0.01 between KA and KA-PTEN (two-way repeated measure ANOVA, n = 13 control, n = 11 KA, n = 8 KA-PTEN). (**b**) The Kaplan-Meier survival curves of control, KA and KA-PTEN mice (n = 13 control, n = 11 KA, n = 7 KA-PTEN) ****P < 0.0001. (**c**) Cardiac function in control, KA and KA-PTEN mice at three and six weeks of age as determined by echocardiography. Scatter dot plots represent the mean ± SD, *P < 0.05, **P < 0.01, ***P < 0.001, ****P < 0.0001 (one-way ANOVA, n = 16 control, n = 11 KA, n = 14 KA-PTEN at the age of three weeks; n = 28 control, n = 17 KA, n = 12 KA-PTEN at the age of six weeks). (**d**) Representative images of hearts (upper images) and heart cross-sections stained with haematoxylin and eosin (lower images) from control, KA and KA-PTEN mice at the age of six weeks. (**e**) Heart weight/body weight ratios of control, KA and KA-PTEN mice at the age of six weeks. Scatter dot plots represent the mean ± SD, *P < 0.05, ***P < 0.001 (one-way ANOVA, n = 19 control, n = 7 KA, n = 9 KA-PTEN). KA: kinase-active IKKβ; KA-PTEN: kinase-active IKKβ with suppressed PTEN; SD: standard deviation. Note: the results for the control and KA groups presented in Figs 1 and 4 are the same.

Next, we investigated the effect of the recovery of insulin signalling on cardiac dysfunction and the survival rate. Surprisingly, KA-PTEN mice showed enhanced body growth and improved survival rates compared with KA mice (Fig. 4a,b). More importantly, echocardiography revealed the significant improvement of cardiac dysfunction in KA-PTEN mice compared with KA mice (Fig. 4c, Supplemental Video 2). Consistent with these results, KA-PTEN mice showed reduced heart dilation (Fig. 4d) and a significant decrease in the heart weight to body weight ratio (Fig. 4e). These results revealed that impaired insulin signalling was involved in the mechanism underlying inflammation-induced cardiac dysfunction and poor survival in KA mice.

### PTEN suppression reduces the number apoptotic cardiomyocytes in KA hearts

**Figure 5:**
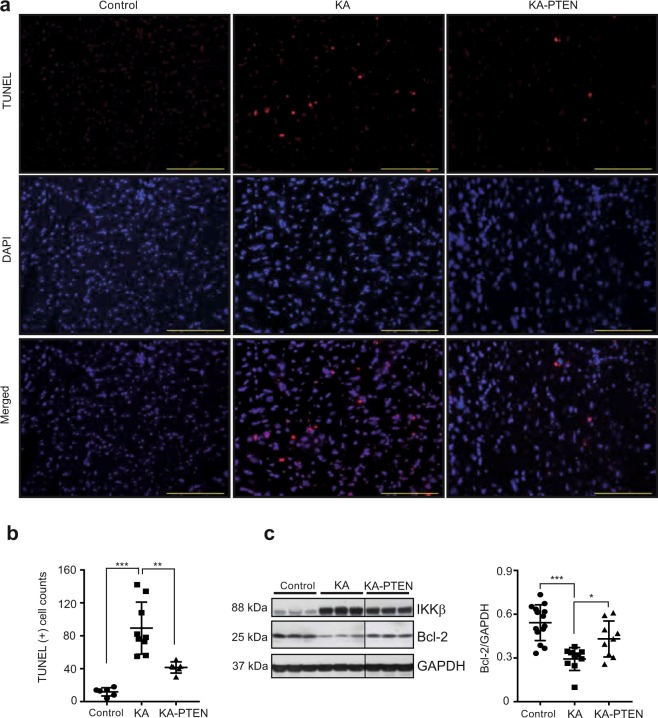
PTEN suppression reduces the number of apoptotic cells in DCMi. (**a**) Representative images of TUNEL assays performed in heart cross-sections from control, KA and KA-PTEN mice at the age of six weeks. Scale bars: 50 μm. (**b**) The graph represents the number of TUNEL-positive nuclei in randomly selected areas in heart cross-sections from control, KA, and KA-PTEN mice. Scatter dot plots represent the mean ± SD, **P < 0.01, ***P < 0.001 (one-way ANOVA, n = 6 mice). (**c**) Representative Western blots and densitometric analyses of Bcl-2/GAPDH expression in heart lysates from control, KA, and KA-PTEN mice at the age of six weeks. There is a line between KA and KA-PTEN samples, which indicates these samples are in separated wells but on the same membrane. Scatter dot plots represent the mean ± SD, *P < 0.05, ***P < 0.001 (one-way ANOVA, n = 14 control, n = 10 KA, n = 9 KA-PTEN). Full-length blots are presented in Supplementary Fig. 5. TUNEL: terminal deoxynucleotidyl transferase dUTP nick end labelling; KA: kinase-active IKKβ; KA-PTEN: kinase-active IKKβ with suppressed PTEN; SD: standard deviation; GAPDH: glyceraldehyde 3-phosphate dehydrogenase.

Apoptosis has been reported as a key mechanism involved in the development of myocarditis and diabetic cardiomyopathy^11,53,54^. In contrast, the activation of insulin signalling is known to induce a pro-survival status^48^. Therefore, we investigated the impact of impaired insulin signalling on apoptosis induced by IKKβ activation. TUNEL staining was performed in cross-sections of hearts from control, KA and KA-PTEN mice. Interestingly, the number of apoptotic cardiomyocytes was remarkably increased in KA hearts compared with that in control hearts, while KA-PTEN hearts showed significantly less apoptosis (Fig. 5a,b). Furthermore, we found that the expression of the apoptotic regulator B-cell lymphocyte (Bcl-2) was significantly downregulated in KA hearts compared with that in control hearts (Fig. 5c). In contrast, Bcl-2 was significantly upregulated in KA-PTEN hearts. These results indicate that the number of apoptotic cardiomyocytes was increased through the downregulation of Bcl-2 in KA hearts and reduced by the suppression of PTEN.

### PTEN suppression alleviates cardiac fibrosis and immune cell infiltration

**Figure 6:**
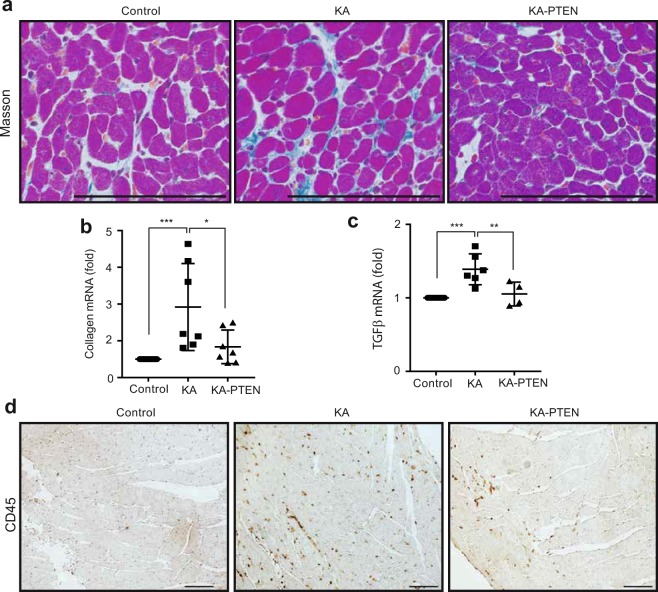
PTEN suppression reduced interstitial fibrosis in DCMi. (**a**) Representative images of Masson staining performed in heart cross sections from control, KA and KA-PTEN mice at the age of six weeks. Scale bars: 50 μm. (**b,c**) Results of qRT-PCR for the mRNA expression of collagen and TGFβ isolated from hearts from control, KA and KA-PTEN mice at the age of six weeks and normalized to the Rn18s mRNA level. Scatter dot plots represent the mean ± SD, *P < 0.05, **P < 0.01, ***P < 0.001 (one-way ANOVA, n = 11 control, n = 7, 6 KA, n = 7, 4 KA-PTEN). (**d**) Representative images of CD45 immunohistostaining performed in heart cross-sections from control, KA and KA-PTEN mice at the age of six weeks. Scale bars: 50 μm. KA: kinase-active IKKβ; KA-PTEN: kinase-active IKKβ with suppressed PTEN; qRT-PCR: quantitative real time-polymerase chain reaction; SD: standard deviation.

Cardiac fibrosis and immune cell infiltration are the main pathological features of myocarditis and DCMi^55^. To examine interstitial fibrosis, Masson trichrome staining was performed in heart sections from mice sacrificed at the age of six weeks. KA hearts showed increased interstitial fibrosis in heart tissue compared with control hearts. In contrast, KA-PTEN hearts showed less fibrosis (Fig. 6a). Furthermore, we found that the mRNA expression of the fibrosis markers collagen 1 and TGF-1β, were significantly increased in KA hearts, and KA-PTEN hearts showed the significant downregulation of both markers (Fig. 6b,c). We also performed IHC in heart cross-sections using CD45, a marker of leukocytes. KA hearts showed a high number of infiltrating leukocytes, while there were fewer infiltrating leukocytes in KA-PTEN hearts (Fig. 6d). These results indicate that the suppression of PTEN reduced interstitial fibrosis and decreased the number of infiltrating immune cells in KA hearts.

## Discussion

The involvement of inflammation in the pathogenesis of myocarditis and DCMi is well known, but the underlying molecular mechanisms have not been adequately explored. For example, the treatment of DCMi with immunosuppressive therapy is not always effective^12,13^, which indicates the contribution of mechanisms other than inflammation. In the present study, we have shown that cardiomyocyte-specific IKKβ activation induced insulin resistance as well as severe cardiac dysfunction, and the reduction of insulin resistance by the suppression of PTEN alleviated cardiac dysfunction and enhanced survival. To the best of our knowledge, this is the first report showing the involvement of impaired insulin signalling in inflammatory cardiac dysfunction.

Myocarditis is a critical disease with high mortality that is induced by virus infection and can be a cause of DCMi, but no effective treatment has been established because of a lack of investigation into the underlying mechanism. Considering the results found in this study, improvement of insulin signalling with anti-diabetic drugs might have a beneficial effect on cardiac dysfunction in myocarditis. Interestingly, Liu *et al*. have recently shown that metformin, a drug that is widely used for type-2 diabetes, has a cardioprotective effect in mice with induced myocarditis via the activation of AMPK signalling^20^. Our data suggest that improved insulin signalling might have contributed to the results of that study.

Numerous epidemiological studies have identified cardiac inflammation and insulin resistance as independent risk factors for the development of heart failure^11,17,56,57^. However, little is known about the relationship between these factors in terms of the mechanism involved in cardiac dysfunction. Although the association between insulin resistance and IKKβ-NF-κB activation has been reported in different types of cells^38–44^, the molecular basis has not yet been revealed in cardiomyocytes. The findings of this study can provide a possible explanation for clinical reports that demonstrated the association of insulin resistance with the elevation of inflammatory markers in non-diabetic patients with heart failure^18,19,58^. The cardiomyocyte-specific overexpression of constitutively active IKKβ has been previously reported by Bärbel Kraut *et al*.^59^. They used an inducible transgenic mouse model with the cardiomyocyte-specific overexpression of constitutively active IKKβ, and this led to defects in heart development and embryonic lethality. On the other hand, the KA mice in our study were born normally, and there was no embryonic lethality. We found that IKKβ expression was not increased in cultured neonatal cardiomyocytes isolated from 1- to 2-day-old KA mice compared with those isolated from control mice (data not shown). The delayed overexpression of IKKβ after birth in mice in our myocarditis model might be the reason for the lack of embryonic lethality and the absence of cardiac defects. In other words, these differences indicate that our myocarditis model is more representative of myocarditis in a clinical setting.

Previous reports have shown that edema is commonly associated with heart failure and leads to weight gain^60^. In contrast, cachexia, which is characterized by the general loss of fat, bone and muscle tissues, has also been considered a serious complication of heart failure^61^. In our study, we observed lower body weight with severe cardiac dysfunction in KA mice, which mimics cachexia in heart failure. The reason which induces the difference is not clear, but considering that KA-IKKβ is constitutively induced (not drug-induced) in our KA mice, the duration of inflammation might be involved in the difference. Given that the lower body weight in KA is considered as cachexia, it is still more interesting that the improvement of insulin signalling was also sufficient to improve cachexia in KA-PTEN mice.

In this study, the recovery of insulin signalling by the suppression of PTEN significantly improved cardiac dysfunction and survival in KA mice, but it should be noted that these effects were not completely rescued. The persistent activation of p65, a major subunit of NF-κB, in cardiomyocytes exacerbates cardiac dysfunction and remodelling in coronary-ligated hearts^62^, and the cardiomyocyte-specific deletion of p65 protects mice from ischaemia-reperfusion injury^63^. The cardiac dysfunction observed in KA hearts is explained by both the activation of IKKβ-NF-κB and impaired insulin signalling.

In conclusion, this study indicates the potential role of impaired insulin signalling in the mechanism underlying inflammation-induced cardiac dysfunction, which suggests that the improvement of insulin signalling can be a target of treatment against myocarditis or DCMi.

## Materials and Methods

### Mice

All animal experiments were approved by the Institute of Laboratory Animals, Graduate School of Medicine, Kyoto University, and performed in accordance with institutional guidelines, which conform to the NIH guidelines. For this study, transgenic mice were crossed into a C57BL/6J background more than 10 times. To activate NF-κB signalling in cardiomyocytes, we generated transgenic mice with constitutively active IKKβ kinase specific to cardiomyocytes (KA), as described previously^31^. IKKβ-KA flox/flox mice were mated α-myosin heavy chain promoter Myh6-Cre^+/−^ mice (The Jackson Laboratory, ME) to generate IKKβ-KA^flox/wt^ Myh6-Cre^+/−^ (KA) mice or IKKβ-KA^flox/wt^ Myh6-Cre^−/−^ (control) mice. To suppress PTEN in KA hearts, we generated heterozygous floxed PTEN mice in the KA transgenic mouse background. First, we produced IKKβ-KA ^flox/flox^ PTEN^flox/flox^ mice, which were then mated with Myh6-Cre^+/−^ mice to generate IKKβ-KA^flox/wt^ PTEN^flox/wt^ Myh6-Cre^+/−^ (KA-PTEN) mice or IKKβ-KA^flox/wt^ PTEN^flox/wt^ Myh6-Cre^−/−^ (control) mice. Mice were born according to Mendelian frequencies. The generated mice were genotyped and screened for the recombinant allele using conventional polymerase chain reaction analysis of tail genomic DNA collected at two weeks of age. Littermate mice were used in all experiments.

### Cardiac imaging

Mice were anaesthetized with 1.5–2% isoflurane via inhalation and maintained in a supine position on a dedicated animal handling platform with limbs fixed for electrocardiogram gating during imaging. Chest hair was removed, and transthoracic echocardiography was performed using a Vevo 2100 system (Visual Sonics, Toronto, Canada) equipped with an MS-400 transducer (30 MHz MicroScan transducer). M-mode recording was performed at the midventricular level. All images were analysed using dedicated software (Vevo 2100 version 1.4). The internal cavity diameters at diastole (LVID;d) and systole (LVID;s) were measured. The percent LV fractional shortening (%FS) and the ejection fraction (%EF) were calculated from M-mode measurements. All measurements were made at the level of the papillary muscles.

### Sample collection

Six-week-old mice were starved for approximately 16 hours and euthanized with an intraperitoneal injection of pentobarbital (70 mg/kg). Blood was collected from the heart, and the vascular system was flushed with cold PBS through the left ventricle. The heart was dissected and photographed. Next, the heart was divided longitudinally into two halves, and the posterior part of the heart was fixed in 4% paraformaldehyde for histological analyses and the anterior part was deep-frozen in liquid nitrogen for further protein and mRNA analysis.

### Histology and Immunohistochemistry

The heart was cut longitudinally and fixed with 4% paraformaldehyde overnight, embedded in paraffin and cut into 4 µm cross-sections. Then, the cross-sections were deparaffinized three times in xylene and dehydrated in 80–90% ethanol for 5 minutes each. They were then stained with haematoxylin and eosin (H&E). For immunohistochemical staining, the cross-sections were boiled in 10 mmol/L citrate buffer for antigen retrieval followed by staining with avidin/biotin. The sections were then exposed to specific primary antibodies overnight at 4 °C. The sections were then incubated with IgG/biotin goat anti-rabbit or goat anti-mouse secondary antibodies for one hour at room temperature, followed by DAB staining. The sections were visualized using a BIOREVO BZ-9000 Keyence microscope.

### TUNEL assay

To evaluate apoptosis in cardiomyocytes in heart tissue, terminal deoxynucleotidyl transferase dUTP nick end labelling (TUNEL) staining was performed using an *in situ* cell death detection kit with TMR red (Roche Molecular Biochemicals, code: 12156792910; Mannheim, Germany), which was used to label the apoptotic cells. In brief, 4 µm-thick formalin-fixed, paraffin-embedded heart sections were deparaffinized and dehydrated as described above and boiled for 2 minutes in 0.1 M citrate buffer, pH = 6, using a microwave. After washing with PBS, the sections were covered with TUNEL reaction mixture for 60 minutes at 37 °C in a humidified atmosphere in the dark. After three washes with PBS, the sections were evaluated under a fluorescence microscope. Positive and negative controls were generated according to the manufacturer’s instructions.

### Extraction of membrane proteins from heart tissue

Mem-PER Plus Membrane Protein Extraction Kit (Thermo Fisher Scientific 89842) was used to extract membrane proteins from heart tissue according to the manufacturer’s protocol. In brief, 20–40 mg of heart tissue was washed with cell wash solution and homogenized in the permeabilization buffer containing protease inhibitor cocktail (Thermo Fisher Scientific 78429) followed by 10 minutes incubation on ice with constant mixing. The homogenized solution was centrifuged at 16,000 × g for 15 minutes at 4 °C to pellet permeabilized cells. Next, the supernatant was carefully removed and the pellets were resuspended in solubilization buffer with pipetting up and down to obtain a homogeneous suspension. Then incubated 30 minutes at 4 °C with constant mixing. The homogeneous suspension was centrifuged at 16,000 × g for 15 minutes and the supernatant was transferred to a new tube for further analysis.

### Western blotting

The left ventricle of the heart was homogenized, and protein extraction was performed on ice using cell lysis buffer (Cell Signalling 9803S) with a protease inhibitor cocktail. After the total protein concentration was measured, the same amount of extracted protein was used for SDS-PAGE immunoblot analysis and transferred to nitrocellulose membranes. Next, the membranes were blocked with 5% non-fat milk or 5% bovine serum albumin (BSA) in Tris buffer solution containing 0.05% Tween-20 (TBS-T) for one hour at room temperature with gentle agitation. The membrane was immunoblotted with primary antibody overnight at 4 °C. The membrane was washed with TBS-T 3 times for 10 minutes each, followed by a one-hour incubation with HRP-conjugated secondary antibody. The blots were visualized by the ECL Western Blotting Detection kit (GE Healthcare). Each band was normalized to the corresponding value of GAPDH as an internal control. Densitometric analysis was performed using ImageJ software.

### Quantitative real-time PCR

Total RNA was extracted from heart tissue using an Ambion RNAqueous kit (AM1912; AM) according to the manufacturer’s instructions. cDNA was generated from the total RNA using the High Capacity RNA-to-cDNA™ Kit (4387406). Quantitative polymerase chain reaction was performed using a QuantiTect SYBR Green PCR Kit and a real-time PCR system (StepOnePlus; Applied Biosystems Japan, Tokyo, Japan). The relative mRNA levels of ANP, BNP, IKKβ, PTEN, TGFβ and collagen were normalized to the level of Rn18s mRNA in the same sample.

### The measurement of NF-κB activity

Hearts were lysed with NE-PER Nuclear and Cytoplasmic Extraction Reagents (Thermo Scientific, Rockford, IL, USA) to generate the nuclear and cytoplasmic extracts. The NF-κB p65 Transcription Factor Assay Kit (Thermo Scientific) was used to detect the active p65 in the nuclear extract according to the manufacturer’s protocol. Briefly, the ELISA plate wells were coated with streptavidin, which bound to a biotinylated NF-κB consensus sequence. Because only the active form of NF-κB (p65) binds to this DNA sequence, nonspecific binding was minimized. The p65-bound consensus sequence was incubated with anti-p65 antibody and then with an HRP-conjugated secondary antibody. A chemiluminescent substrate was added to the wells, and the resulting signal was detected using a luminometer.

### Antibodies and reagents

The antibodies used included anti-IKKβ (Millipore, Billerica, MA, USA), anti-GAPDH, anti-IRS-1, anti-phospho-IRS-1(s318), anti-Bcl-2, anti-AS160, anti-phospho-AS160 (s588), anti-Akt, anti-phospho-Akt (s473), anti-phospho-p65 (s536), anti-Na-K-ATPase, anti-mouse IgG HRP-linked and anti-rabbit IgG HRP-linked antibodies, (Cell Signaling Technology, Boston, MA, USA), anti-Glut4, anti-p65 (Santa Cruz), goat anti-rabbit IgG, Alexa Fluor 488 goat anti-mouse IgG, Alexa Fluor 594 donkey anti-goat IgG, and Alexa Fluor 488 donkey anti-goat IgG (Invitrogen, Foster City, CA, USA). Mem-PER Plus Membrane Protein Extraction Kit and NE-PER Nuclear and Cytoplasmic Extraction Reagents (Thermo Fisher Scientific) were also used.

### Statistical analysis

The experimental results are expressed as the mean ± SD. The groups were compared using a 2-tailed Student’s t test or one- and two-way repeated measure analysis of variance (ANOVA), followed by Tukey’s test. P values of <0.05 were considered statistically significant. The statistical analysis was carried out using GraphPad Prism 7.

## Supplementary information

Supplemental video1

Supplemental video2

Supplementary Figures and Video legends

## Data Availability

All additional data from the experiments are provided in Supplementary Information.
