# Supplementary material for: Improvement of insulin signalling rescues inflammatory cardiac dysfunction
Source: Sci Rep. 2019 Oct 15;9:14801. doi: 10.1038/s41598-019-51304-8 (PMC6794250; doi:10.1038/s41598-019-51304-8)
Supplement: Supplementary file 3 — Supplementary Figures and Video legends [file 41598_2019_51304_MOESM3_ESM.pdf]

Title: Improvement of insulin signalling rescues inflammatory cardiac dysfunction

Authors: Isehaq Al-Huseini[1], Masayuki Harada[1], Kiyoto Nishi[1], Dat Nguyen-Tien [1], Takeshi Kimura[1], Noboru Ashida[1] *

Affiliations:

1Department of Cardiovascular Medicine, Graduate School of Medicine, Kyoto University, Kyoto, Japan

Current address of Isehaq Al-Huseini; Department of Physiology, College of Medicine and Health Sciences, Sultan Qaboos University, Muscat, Oman

Current address of Kiyoto Nishi; Mitochondria and Metabolism Center, Department of Anaesthesiology and Pain Medicine, University of Washington, Seattle, WA98109, USA

Current address of Dat Nguyen-Tien; Department of Molecular Immunology and Inflammation, Research Institute, National Center for Global Health and Medicine, 1-21-1 Toyama, Shinjuku-ku, Tokyo 162-8655, Japan.

*Corresponding author: Noboru Ashida M.D., PhD; E-mail: nashida@kuhp.kyoto-u.ac.jp; Department of Cardiovascular Medicine, Graduate School of Medicine, Kyoto University, Kyoto, Japan. Address: 54 Kawa-hara-cho, Shogoin, Sakyo-ku, Kyoto, Japan, 606-8507. Telephone: +81-75-751-3195. Fax: +81-75-751-3203

Supplementary information Figure 1.

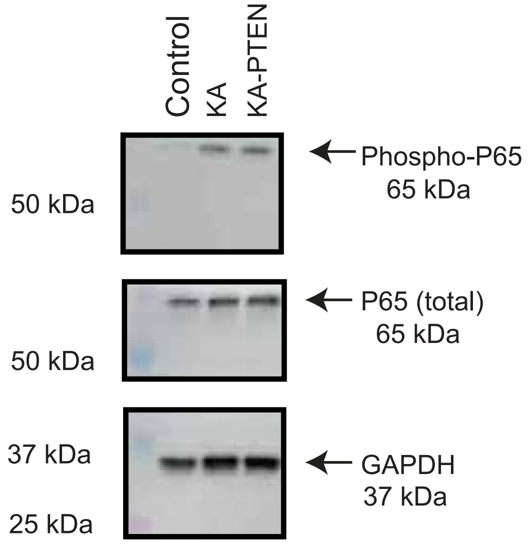

Supplementary figure 1. Full length immunoblots of anti-phospho-PP65, anti-P65(Total) and anti-GAPDH as loading control.

Supplementary information figure 2.

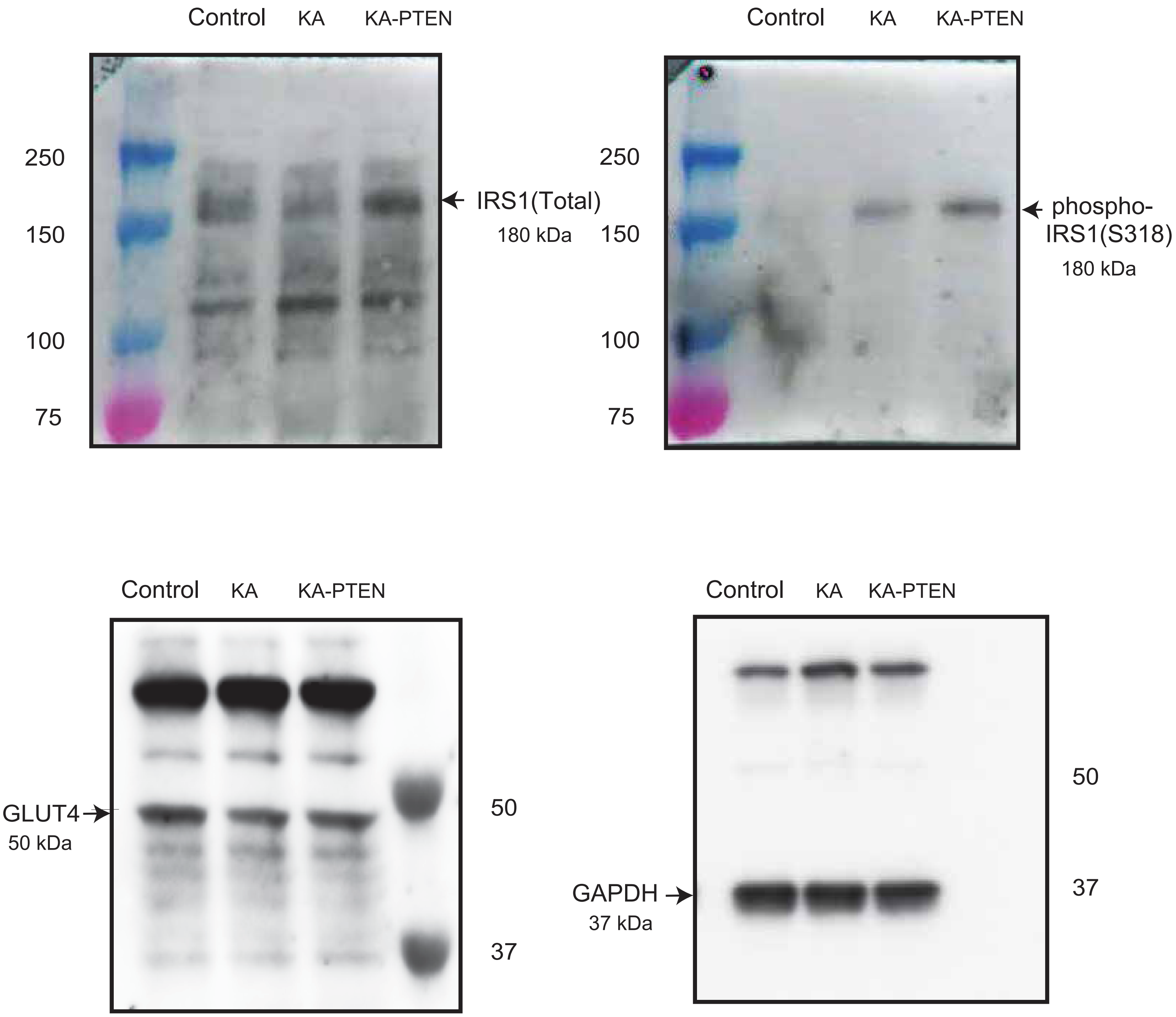

Supplementary figure 2. Full length immunoblots of anti-phospho-IRS1 (serine318) and anti-IRS1 (total), anti-Glut4 and anti-GAPDH as loading control.

Supplementary information figure 3.

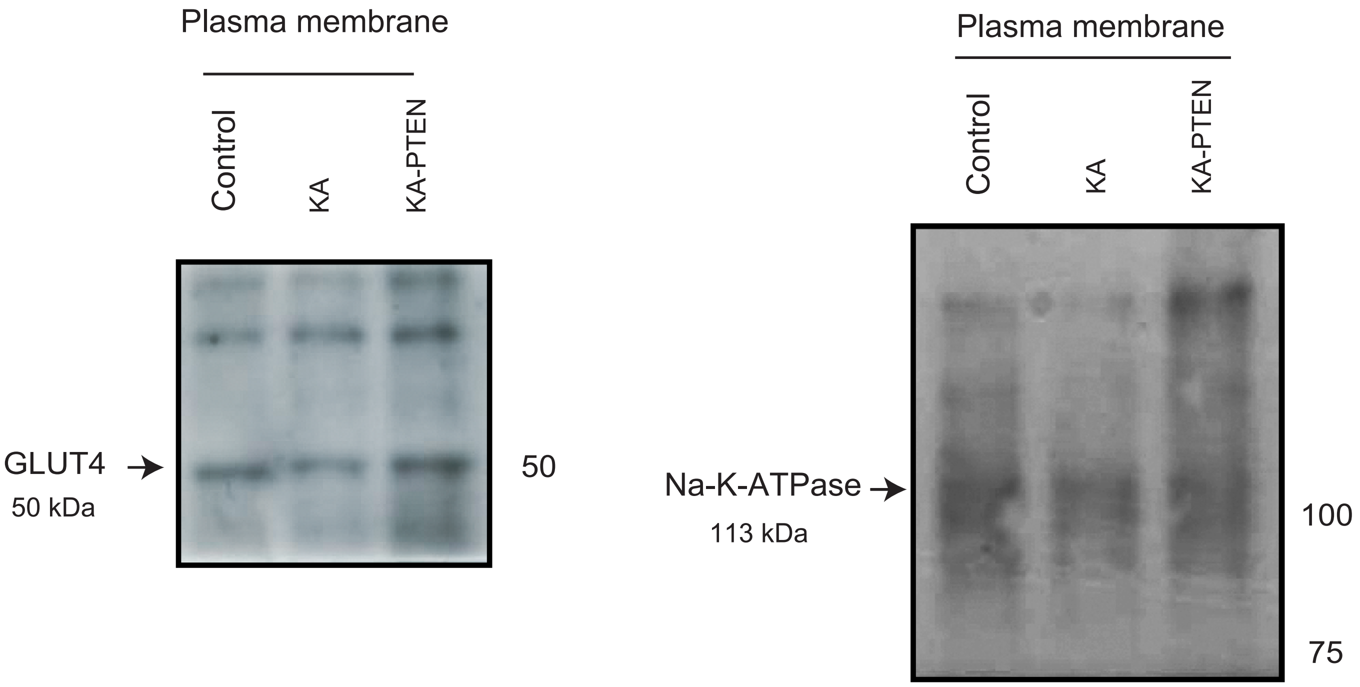

Supplementary figure 3. Full length immunoblots of anti-Glut4 and anti-NA-K-ATPase.

Supplementary information figure 4.

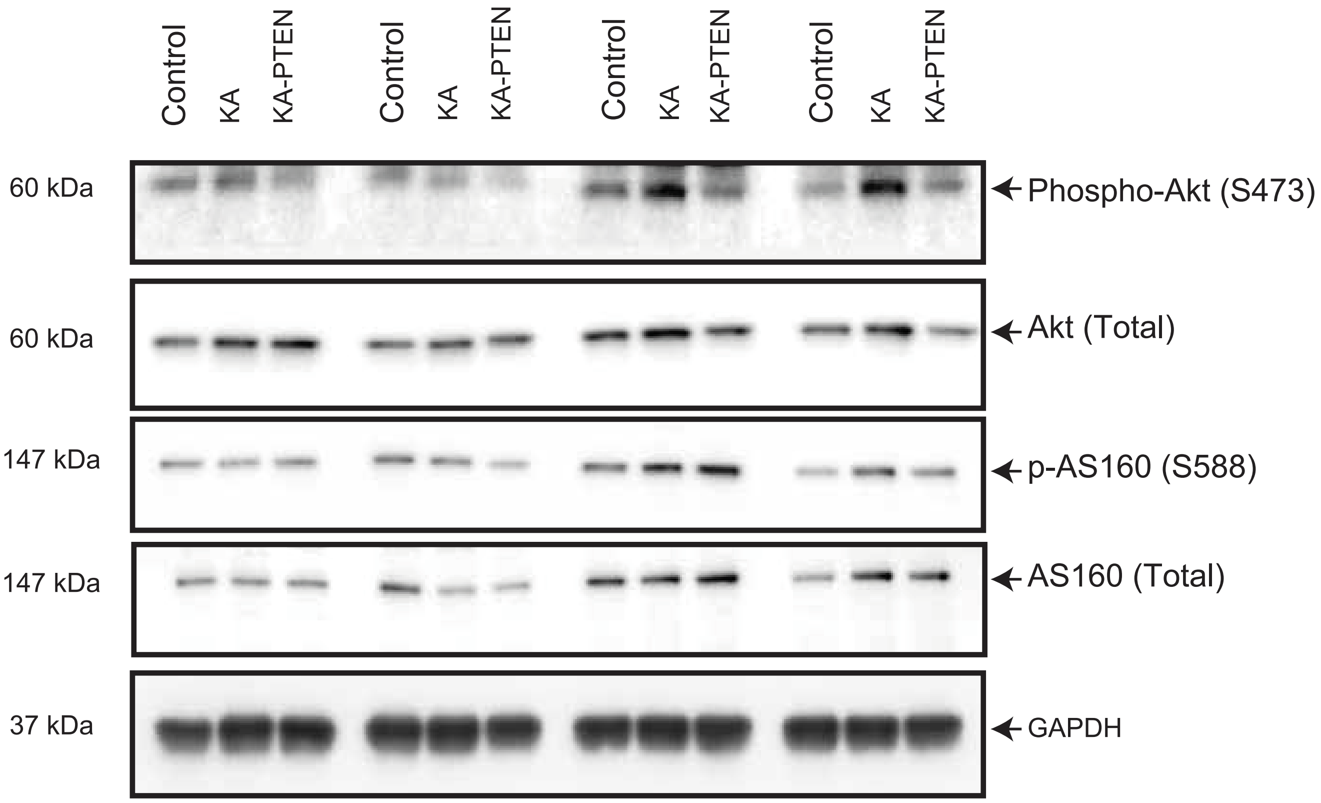

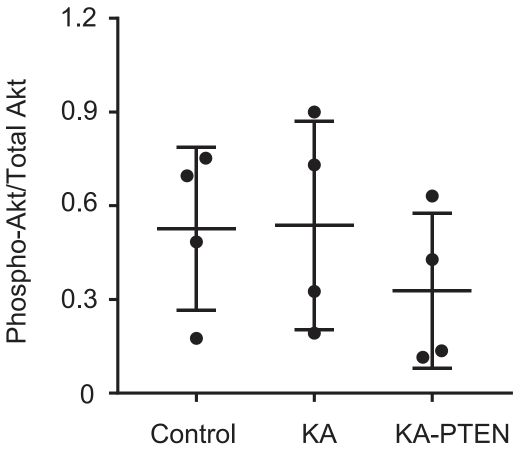

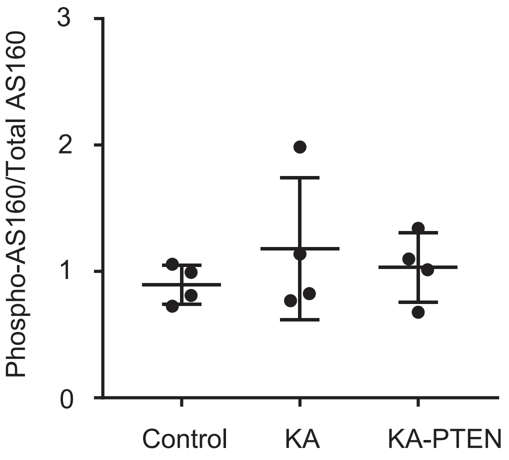

Supplementary figure 4. Full length immunoblots of phospho-Akt (S473), anti-Akt (Total), anti-phospho-AS160 (S588) and anti-AS160 (Total) and anti-GAPDH as loading control. Densitometric analyses of phospho-Akt ser473/total Akt and phospho-AS160 Ser588/total AS160 in heart lysates. Scatter dot plots represent the mean ± SD

Supplementary information figure 5

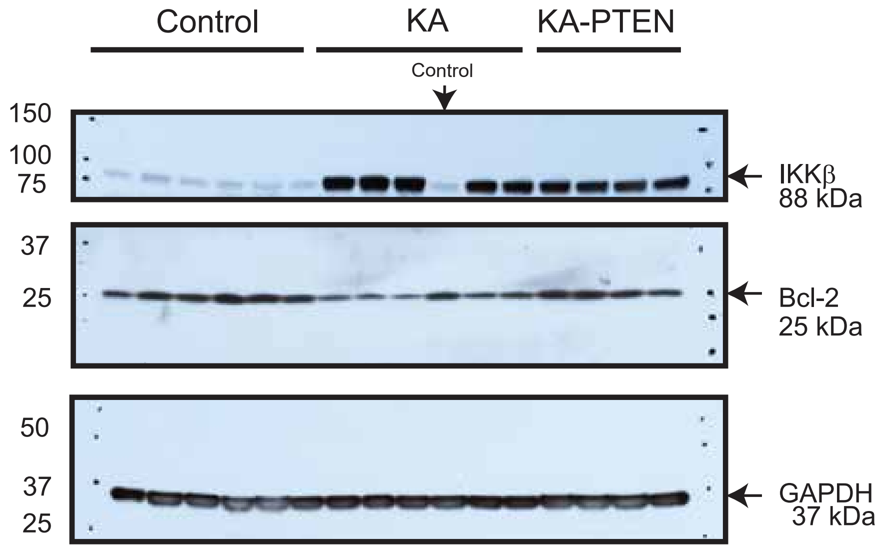

Supplementary figure 5. Full length immunoblots of anti-IKKβ, anti-Bcl-2 and anti GAPDH as loading control.

Supplemental Video 1. Representative echocardiography video taken at the mid-ventricular short-axis of control and KA mice at the ages of three and six weeks.

Supplemental Video 2. Representative echocardiography video taken at the mid-ventricular short-axis of control and KA mice (the same videos that are presented in Supplemental Video 1) and also of KA-PTEN mice at the ages of three and six weeks.
